# Supplementary material for: A qualitative exploration of the experiences of pregnant women living with obesity and accessing antenatal care
Source: PLoS One. 2024 May 3;19(5):e0302599. doi: 10.1371/journal.pone.0302599 (PMC11068194; doi:10.1371/journal.pone.0302599)
Supplement: Attachment — Submitted filename: Final Response to reviewers comments .docx [file pone.0302599.s001.docx]

Response to reviewers’ comments

Reviewer 1

| Thank you for giving me the opportunity to read and review this interesting and important manuscript about how pregnant women with obesity have experienced the antenatal care.  The study is important as weight related health in pregnancy affect generations, while existing interventions seem insufficient. The authors present findings that confirm earlier study results but also describes new angles, such as how willingness to enroll in a study may be driven by a perceived lack of existing support.  The method is described in a clear fashion and the writers present and discuss their findings in a relevant way. The quotes are well-chosen and support the results. I enjoyed reading it and believe it will be ready for publication after dealing with some minor, but important, issues and language revisions. | Many thanks for your positive feedback |
| --- | --- |
| Areas of improvement In the discussion section, I suggest the authors mention the limitation that the interviews were performed more than 10 years ago and thus pregnant women with obesity may have different experiences today, as the treatment and attitudes towards obesity may have changed. For the same reason, I suggest that the conclusion section is slightly adjusted overall by using past tense to indicate that the conclusions are based on how women experienced the care over 10 years ago. For example, line 607-608, where it says ”…it is evident that there is considerable…” could rather read “…it is evident that there was considerable…”. | This has been addressed in the newly added limitations section and past tense terms added in conclusion. |
| Language The language is well-written. However, throughout the entire manuscript, including in tables, there are about 95 errors where space between words is lacking, such as “isimportant” (line 104) and “asinevitable” (line105) and so on. There are also a large number of missing spaces after commas, full stops and references such as in line 80 "collegues10found" or in line 81 “(n=92)reported” and “care,with”. The need for corrections should be easily found by using the grammar and spelling function in Word | Apologies, this is due to an auto-typo that has occurred during the saving process.  The file is now saved under the same format. |
| .Besides these spelling error I suggest the following language edits: Line 39 (introduction). Remove either “a” or “the” in the first sentence. | Removed ‘a’ |
| Line 53 Are women really advised to lose weight intrapartum, as in during labour? If not please clarify or perhaps remove the word intrapartum. | The word ‘intrapartum’ removed |
| Line 92 This sentence left me confused. Is there perhaps something missing at the end? Something like: “…if they did not attend the weight management course”. Please clarify. | Now amended to: Understanding the experiences of living with obesity, enables HCP to guide pregnant women towards more positive behaviour changes and to foster more meaningful discussions with regards to weight. However, perceptions of weight stigma, insensitivity, or feelings of being judged by HCPs may hinder such conversations  See page 4 |
| Line 147, Table 1. I wonder about the purpose of pseudonyms in the table. Unless they are used instead of numbers under the quotes, the column could be removed. | Pseudonyms column removed |
| Line 450-453 (discussion section). The sentence left me confused. It starts with “similar to” and continues with “but in contrast”. If the words “but in contrast” is removed it seems to make sense but I am not sure that is what the authors want to point out. Please clarify the meaning. | This sentence has been amended to Perceptions of weight bias in HCP, towards the general patient population living with overweight and obesity, have been reported in a number of UK studies ^25–27^. Similarly, pregnant women living with obesity have also experienced weight stigma from HCPs.  See page 21 |
| Line 491-492 (discussion section) The statement “Midwives are often reluctant…” could use a reference, for example: Shame and avoidance as barriers in midwives' communication about body weight with pregnant women: A qualitative interview study. Christenson, A., Johansson, E., Reynisdottir, S., Torgerson, J., &Hemmingsson, E. (2018). Midwifery, 63, 1–7. <https://doi.org/10.1016/j.midw.2018.04.020> | Many thanks for the excellent reference that is appropriate to the theme, it has been included as a reference  See page 23 |
| Other comment I really liked that the authors had put examples of interview questions in a table (instead of me having to look for them in a supplementary material). | Many thanks for your feedback and comments with regards to our paper, we hope that we have addressed the issues raised to your satisfaction. |

Reviewer 2

| Thank you for the opportunity to review ‘A qualitative exploration of the experiences of pregnant women living with obesity and accessing antenatal care’. This paper provides an interesting insight into the antenatal experience of women with high BMI. Overall the paper is well written and provides useful recommendations for future interventions. | Many thanks for your positive feedback and helpful comments. |
| --- | --- |
| The decision to omit strengths and weaknesses of the study in the discussion is regrettable, as it should be noted that the particular demographic of women interviewed in the study had already volunteered for a study on gestational weight gain, so observations and recommendations may be skewed by this association, which is not acknowledged in the paper. It is not clear why women with BMI >35kg/m2 were included when reference to either BMI >30kg/m2 or BMI >40kg/m2 are noted in other parts of the paper as having more significant influence on care provision. Further clarity is also needed on the participant group, whether they were pregnant at the time of interview or postnatal, and if interviews were postnatal how many weeks post pregnancy were the women? Please also provide consistent figures in how many were recruited/lost to follow up etc.  Attention should be paid to grammatical and structural errors in this paper which are noted below. Overall, I believe this paper makes a valuable contribution to the literature on this topic, however the following concerns should be addressed prior to publication: | We have now addressed strengths and limitations.  All other comments are addressed below: |
| Abstract: Discussion of midwives beliefs and perceptions does not seem justified in the abstract as this paper is only exploring experiences of women. Sentence "This study explored the experiences of accessing antenatal care in pregnant women living with obesity." - antenatal pregnant care in pregnant women does not make sense | We have reduced some of the discussion of midwifery beliefs in the abstract.  The wording has been changed to clarify certain points. |
| Line 23 Repeated issue throughout this paper with words joined together. In this instance, foradvising and risksthroughout | Apologies, this is due to an auto-typo that has occurred during the saving process.  The file is now saved under the same format. |
| Line 39 Pregnancy is often a the time – remove a | Removed ‘a’ |
| Line 46 Growing emphasis on not for | Amended to ‘on’ |
| Line 53 Weight loss is not recommended intrapartum in the guidelines referenced. Provide supporting evidence if suggesting weight loss during pregnancy is advised. | The word ‘intraparum’ has been removed. |
| Line 60 Reference 7 is exploring mothers’ experience of discussions on weight, not midwives experience, it does not seem an appropriate reference for a statement regarding midwives attitudes to weight counselling. There are a number of empirical sources to refer to on this topic. | We agree that there are a number of studies regarding this topic; however reference 7 is a well-known study in the UK. The focus of the paper is women’s experiences, but the beliefs and attitudes of midwives have a part to play in this experience. |
| Line 82 Source of weight stigma not cause | Wording amended to ‘source’ |
| Line 87 Review this sentence for clarity | We have changed this sentence to:  “Many women reported being upset on receiving the referral letter for weight management services; either because the referral had not been discussed with them (by the HCP who made the referral), or they did not receive enough information about it and so they felt that the service did not meet their needs.’  See page 4 |
| Line 91 What makes this more worrying? | Wording amended to: Further to this,  See page 4 |
| Line 123 I would encourage use of primary references such as Braun and Clarke, rather than secondary references to justify this approach to data analysis | Braun and Clarke 2006;2022 references included within text  See page 5 |
| Line 124 Up to how many weeks postnatally were women included? | Women were included who were between 12 and 24 months postnatal – this has been added.  See page 5 |
| Line 128 Review this sentence for clarity | Wording amended to This study enabled the personal perspectives and experiences of women living with obesity during pregnancy to be better understood.  See page 5 |
| Line 141 Were all women pregnant or were some postnatal? If including postnatal women, what time-periodpost-birth were these interviews conducted? | Wording amended to provide clarity  See page 5 |
| Line 141 Why was BMI >35kg/m2 chosen when you have noted classification of obesity and recommendations regarding obesity start at BMI 30kg/m2? | BMI ≥35 kg/m^2^ was selected to match the inclusion criteria of Fit For Birth Plus. Citations to related papers have been added ( 17, 18).This following has also been added. ‘At the time of data collection BMI ≥35 kg/m^2^was the cut-off for accessing more specialist antenatal care at the hospital.’  See page 6 |
| Line 161 Why were 9 women excluded from recruitment? | There is a comment in the text relating to 9 women who had left the area and were no longer contactable  See page 8 |
| Line 164 78 – 9 – 35 – 15 = 19 or 69-9-35-15 = 10. The figures do not add up. Please review. | Data double checked and unresponsive women amended to 36.  See page 8 |
| Line 178 Clarity required regarding approach to thematic analysis. Was it Braun and Clarke’s approach to Thematic Analysis as suggested in the table? Though data may have been analysed prior to Braun and Clarke’s 2022 Thematic Analysis textbook being printed, it may be worth clarifying explicitly that you used the 2006 approach and why you opted for that instead of the Reflexive Thematic Analysis of 2022 as this paper will be published >2 years after publication of the new Braun and Clarke approach. | This research project, ethical approval and research plan was put in place with reference to Braun and Clarkes Thematic Analysis, 2006.  The data analysis and therefore write up of this article has been true to the analysis as planned utilising the original 2006 TA steps.  We are fully familiar with the nuanced changes applied to Braun and Clarkes 2022 updated reflective thematic analysis, though note that the previous 2006 TA version is still applicable. As such the description of the analysis stands as stated.  See page 8 |
| Line 183 Full stop rather than semi-colon more appropriate here | Amended |
| Line 182 Review this sentence for clarity | Reviewed and amended (MC is now after author, not nutritionist)  See page 9 |
| Line 187 The word experience is used repeatedly in this sentence, perhaps consider alternative word? | Reviewed and amended to:  ‘All authors are parents and had various experiences of encountering antenatal and postnatal care services. These personal experiences were deemed helpful to be able to empathise and understand the women’s perspectives.’  See page 9 |
| Line 230 If this is a study of antenatal care, why is there reference to the ward? Was it an antenatal ward? | Yes, this could be an antenatal ward – women are sometimes admitted if there are concerns about their health or their pregnancy.  See page 12 |
| Line 242 Spelling error of ‘you’re’ | This has been amended to ‘you’re’. |
| Line 262 Full stop after them should be removed | Removed and a comma is added instead. |
| Line 274 More context needed for why bad weather would impede contact with a midwife? | Bad weather can affect the running of public transport (this has now been clarified  See page 13 |
| Line 276 Is ‘in the dark’ a quote from a participant? | Yes – this is now indicated by italics  See page 14 |
| Line 317 Are aquanatal classes provided within healthcare settings? If not, how are these examples specific to experiences within healthcare settings? | The classes are not provided within the healthcare setting, but were encouraged by midwives as part of their antenatal care, so still relevant here.  See page 15 |
| Line 358 Review use of comma alongside full stop and lack of capitalisation | Full stop is removed |
| Line 398 Which participant is this quoted from? | R6 identified as participant  See page 19 |
| Line 432 Reconsider use of ‘one’ in this sentence as healthy weight is a range, not a singular figure | ‘To achieve one’, has been changed to ‘achieve this’.  See page 20 |
| Line 441 contribute should be contributes | Amended. |
| Line 445 Severe? Or several? | Several – this has been amended. |
| Line 450 This sentence does not make sense | This has been amended. |
| Line 460 See also the systematic review by Puhl (2020) regarding weight related terminology, note that the term obesity, with or without person-first language, is widely considered stigmatising by the affected group | Thank you for this suggestion – this reference is now added.  Puhl, R.M., 2020. (ref 29)  See page 22 |
| Line 465 Worth noting that in the field of Fat Studies fat is not considered pejorative and has been reclaimed, however agree that it was used negatively in these specific interactions | We agree. |
| Line 472 Review this sentence for clarity | This is now amended to:  ‘People living with obesity, (including pregnant women), demonstrated a reluctance to discuss aspects of their weight with HCPs, and equally, HCPs expressed discomfort at having to raise issues around weight with patients.  See page 22 |
| Line 476 Review this sentence for clarity ‘why the referral was to weight management support was necessary’ | This is amended to:  ‘why the referral to weight management support was necessary’,  See page 22 |
| Line 477 A negative experience described by whom? Or is it that after women had a negative interaction they were reluctant to return? | Amended to‘if they had had a negative experience regarding...etc.’  See page 22 |
| Line 481 It is ‘never’ neutral? Is this supported by evidence? | This is similar to Freire’s philosophy, that education is never neutral – so we have added this in  Ledwith, M., 2016. Press (32)  See page 22 |
| Line 485 Do HCP have any training regarding weight stigma? Let alone ongoing? | Unlikely, we have removed the word ‘ongoing’.  See page 23 |
| Line 491 What training currently exists? Line 492 Support this statement with evidence | We are unaware of any details regarding current training.  References (8, 33) has been added in support.  See page 23 |
| Line 497 ‘Is’ should be added to this sentence - ‘is not offered’ | ‘Is’ now added |
| Line 503 There is an undergraduate training programme for pregnant women? | The undergraduate programme is for student midwives, this has been clarified.  See page 24 |
| Line 514 Many reported positive experiences and many reported negative, can you be more specific is there a figure? Did women who reported negative experiences also report some positive experiences or was it definitively one way or the other? | It is not usual to apply figures to qualitative research, it is actively discouraged in TA (Braun and Clarke). Specific figures have not therefore been included. Nevertheless, a mixture of experiences was reported. |
| Line 516 Is this sentence referring to women in the study or patients more generally? It’s confusing to go from talking about participants in the study to patients generally without qualification. | Wording has been revised to‘Research studies have reported delays or avoidance of appointments or interactions with healthcare services due to weight stigma where HCPs have demonstrate weight bias, leading to inconsistencies in care’  See page 24 |
| Line 524 Of should be added to this sentence– ‘only a quarter of’ | ‘of’ is now added.  See page 24 |
| Line 530 Negative stereotypes lead to perceptions, consider rewriting this sentence to aid clarity in why terms such as lazy are reported – are these negative perceptions of people with high BMI or are these terms used in consultation… | These are negative perceptions of people with higher BMI; they relate to perceptions of poor self-management behaviours. This has been clarified and an additional reference is now added:  Mulherin, K., Miller, Y.D., Barlow, F.K., Diedrichs, P.C. and Thompson, R., 2013. (39)  See page 25 |
| Line 533 Knowledge of what? Knowledge of obesity in what context? Clinically? | Is amended to:  However, it was determined that knowledge regarding the aetiology and risk factors associated with obesity were relatively low in some HCPswith the belief that obesity is in the control of the individual still persisting.’  See page 25 |
| Line 536 Reference needed for sentence starting with ‘The belief..’ | A reference has been added for this:  Teixeira, F.V., Pais-Ribeiro, J.L. and da Costa Maia, Â.R.P., 2012. (40)  See page 25 |
| Line 545 I encourage review of Fair &Soltani (2021) Meta review of lifestyle interventions to reduce gestational weight gain in this context. If encouraging midwives to provide comprehensive diet and weight management advice, is there clear evidence of benefit to maternal and/or fetal outcomes? | Thank you for highlighting this text, while Fair & Soltani did not observe any benefits to maternal and fetal outcomes from pregnancy interventions, current UK guidelines expect midwives to have conversations with ALL pregnant women about diet and weight; furthermore, pregnant women want this information from midwives as part of their package of care.  We have added reference to the NICE guidance here to clarify this.  NICE 2021 Antenatal care. Available at: <https://www.nice.org.uk/guidance/ng201>  See page 25 |
| Line 545 Consideration needs to given as to whether UK or US spelling will be used in this paper. Both conventions noted, including behaviour and minimized. Opt for one convention and ensure consistency. | We are using UK spelling & we have amended this, thank you. |
| Line 548 How were women able to differentiate between pregnancy or obesity exacerbating asthma and sciatica? | This was their perception that obesity exacerbated these conditions.  This has been amended to reflect this.  See page 25 |
| Line 550 Evidence suggests rather than Indications are. Alternatively, what indications? | This has been amended to ‘Evidence suggests’  See page 25 |
| Line 588 Review use of commas in this sentence | Comma has been removed. |
| Line 591 Consider use of word variability in this context | This has been changed to variety.  See page 27 |
| Line 596 And/or | Amended. |
| Line 611 Is it possible to make this statement given no midwives were interviewed as part of this study? | This has been removed as it does not relate to the findings of this present study.  See conclusion. |
| Line 618 Review this sentence for clarity | This has been amended to:  ‘There is an urgent need to ensure that pregnant women living with obesity have access to enhanced care, which reflects their risk. Moreover multidisciplinary training for HCPs is also needed, which addresses the concerns of those living with obesity and seeks to improve healthcare delivery, based on some of the experiences of pregnant women, living with obesity, as outlined in this study’.  See conclusion |
