# Supplementary material for: A qualitative exploration of the experiences of pregnant women living with obesity and accessing antenatal care
Source: PLoS One. 2024 May 3;19(5):e0302599. doi: 10.1371/journal.pone.0302599 (PMC11068194; doi:10.1371/journal.pone.0302599)
Supplement: Attachment — Submitted filename: Review 2.docx [file pone.0302599.s002.docx]

Dear authors,

I consider your amendments to be satisfactory and after making two further minimal changes (which does not need another round of review) I believe the article is ready for publication.

The changes are:

Line 391

To be consistent throughout your quotes, you should remove the full stop after the quotation mark and before [R6]. It now says: Watchers”. [R6] while the other quotations do not have a full stop there.

Line 511 Please remove the apostrophe after the word “care”
